# Mother-child bed-sharing trajectories and psychiatric disorders at the age of 6 years

**DOI:** 10.1016/j.jad.2016.08.054

**Published:** 2017-01-15

**Authors:** Iná S. Santos, Aluísio JD Barros, Fernando C. Barros, Tiago N. Munhoz, Bianca Del Ponte Da Silva, Alicia Matijasevich

**Affiliations:** a Programa de Pós-graduação em Epidemiologia, Universidade Federal de Pelotas, Pelotas, RS, Brazil; b Departamento de Medicina Preventiva, Universidade de São Paulo, São Paulo, SP, Brazil

**Keywords:** Cohort studies, Mental health, Sleep, Epidemiology

## Abstract

**Background:**

Little is known about the effect of bed-sharing with the mother over the child mental health.

**Methods:**

Population-based birth cohort conducted in Pelotas, Brazil. Children were enrolled at birth (n=4231) and followed-up at 3 months and at 1, 2, 4, and 6 years of age. Bed-sharing was defined as “habitual sharing of the bed between the child and the mother, for sleeping, for part of the night or the whole night”. Trajectories of bed sharing between 3 months and 6 years of age were calculated. Mental health was assessed at the age of 6 years using the Development and Well-Being Assessment instrument that generates psychiatric diagnosis according to ICD-10 and DSM-IV criteria. Odds ratios (OR) with 95% confidence intervals were obtained by multivariate logistic regression.

**Results:**

3583 children were analyzed. Four trajectories were identified: non bed-sharers (44.4%), early-only (36.2%), late-onset (12.0%), and persistent bed-sharers (7.4%). In the adjusted analyses persistent bed-sharers were at increased odds of presenting any psychiatric disorder (OR=1.7; 1.2–2.5) and internalizing problems (OR=2.1; 1.4–3.1), as compared to non bed-sharers. Among the early-only bed-sharers OR for any psychiatric disorder was 1.4 (1.1–1.8) and for internalizing problems 1.6 (1.2–2.1).

**Limitations:**

Although the effect of bed-sharing was adjusted for several covariates including the family socio-economic status, maternal mental health and excessive crying, there was no information on maternal personal reasons for bed-sharing. Mothers that bed-share intentionally and those that bed-share in reaction to a child sleep problem may have a different interpretation of their children behavior that may bias the study results.

**Conclusion:**

Bed-sharing is a common practice in our setting and is associated with impaired child mental health at the age of six years.

## Highlights

• Sharing parents’ bed in infancy and childhood is a common caring practice in several cultures.
• Persistent and early-only bed-sharers presented increased odds of internalizing problems at 6 years when compared to non bed-sharers.
• Outcomes of bed-sharing may depend on maternal reasons for bed-sharing with their children.

## 1 Introduction

Sharing parents’ bed in infancy and childhood is a common caring practice in several cultures (Mindell et al., 2010). Among the most widely reported factors associated with a greater prevalence of bed-sharing are socioeconomic factors like lower family income, lower maternal age, lower maternal education, and non-white skin color (Colson et al., 2013, Blair et al., 2010). Bed-sharing can be intentional or reactive. Intentional bed sharing refers to parents who intended to bed share and do so from early infancy onward (Cassels, 2013). Parents may bed-share with the child intentionally due to cultural beliefs, breastfeeding facilitation, parental ideology, parental own sleep experiences, convenience, anxiety, child safety, parent and child emotional needs, better infant sleep, unavailability of other beds, enjoyment, physical proximity to the infant, and better caregiving (Mileva-Seitz et al., 2016). On the other hand, bed-sharing can be reactive in which the parent starts to bring the child to bed after the age of one year in response to problematic circumstances typically in response to bad sleeping patterns by the child (Mileva-Seitz et al., 2016, Cassels, 2013).

Systematic reviews focusing on harms and benefits of bed-sharing (Horsley et al., 2007, Das et al., 2014) reported a positive association between bed-sharing and increased duration of breastfeeding although the issue of causality (whether bed sharing promotes breastfeeding or whether breastfeeding promotes bed-sharing) remains unclear. Also, infants who bed-share have an increased number of awakenings when compared with solitary-sleeping infants, and individual awakenings are shorter in the bed-sharers than in the solitary sleepers (Horsley et al., 2007), suggesting an infant’s ability to rouse that may be protective against sudden infant death syndrome. On the other hand, there may be association between bed-sharing and sudden infant death syndrome among smokers (Horsley et al., 2007). The findings from two large case-control studies showed that bed-sharing is inappropriate if parents consume alcohol, take drugs or smoke, or if the infant is pre-term (Blair et al., 2014).

Studies reporting other harms or benefits of bed-sharing, like the risk of hospitalizations (Ngale et al., 2013), cognitive and behavioral problems (Barajas et al., 2011, Okami et al., 2002, Madansky and Edelbrock, 1990), psychiatric symptoms (Kaymaz et al., 2014), and psychosexual development (Jain et al., 2011) in offspring are scarce at the literature. This study aimed to describe bed-sharing trajectories from 3 months to 6 years of age and to investigate the association between bed-sharing trajectories and mental health at the age of 6 years among children from a population-based birth cohort. The primary hypothesis was that bed-sharing was associated with increased prevalence of mental symptoms at the age of 6 years.

## 2 Methods

This study was carried out in Pelotas, the third most populous city in the southern state of Rio Grande do Sul, located 270 km from Porto Alegre, the capital city of the state, and 130 km from the Uruguayan border. With an estimated population in 2015 of 342,873 inhabitants, the major economic activities of the municipality are agriculture, trade and services. The city has five universities, three private and two public (Prefeitura Municipal de Pelotas, 2016). Economically, when compared to the state of Rio Grande do Sul, the Pelotas region is relatively poorer. In 2012, its per capita GDP was equivalent to 65% of the state average (Ibge - Instituto Brasileiro De Geografia E Estatística, 2016b, Seplan - Secretaria Do Planejamento, 2016). In terms of health, in 2013, the infant mortality rate in the state of Rio Grande do Sul and in Pelotas was 10.5 and 9.9 per thousand live births, respectively (Ibge - Instituto Brasileiro De Geografia E Estatística, 2016a, Secretaria Estadual Da Saúde, 2016).

In 2004, a birth cohort study attempted to enroll all births to mothers resident in the urban area of Pelotas. Eligible mothers – those living in the urban area of Pelotas municipality and in the Jardim América neighborhood – were interviewed using a standardized, pre-coded questionnaire. Mothers were interviewed at the hospital (there were five hospitals with maternity ward in 2004 at the city) soon after delivery regarding demographic, socio-economic, behavioral and biological characteristics, reproductive history, and health care services utilization. Non-hospital deliveries were also included in the cohort, since mothers normally sought a maternity ward after delivery, and were thus recruited to the study at this stage. The non-response rate at recruitment was below 1%. A total of 4231 live births were enrolled in the cohort. Follow-ups including the entire cohort sample were done at home at mean ages 3.0±0.1, 11.9±0.2, 23.9±0.4 and 49.5±1.7 months, and at a research clinic at 6.8±0.3 years, with follow-up rates between 90% and 96%. Such a high retention rates are the result of concerted effort to collect contact information data from families, a process that was facilitated by the large expansion of telephone services, especially mobile phones in the country in the last years. A detailed description of the Pelotas 2004 Birth Cohort Study methodology is given elsewhere (Santos et al., 2014).

Bed-sharing was defined as ‘habitual sharing of the bed between the child and the mother, for sleeping, for part of the night or the whole night’. A semi-parametric, group-based approach (a specialized form of finite mixture modelling designed to identify rather than assume groups or clusters of individuals following similarly developmental trajectories) (Nagin, 2005, Nagin and Tremblay, 1999) was used to identify the different patterns of bed-sharing reported by mothers from 3-month until the 6-year follow-up. A polynomial function was used to model the relationship between bed-sharing and age (Nagin and Tremblay, 1999, Nagin, 2005, Nagin and Odgers, 2010). The models were estimated with the Stata procedure “traj” (Jones and Nagin, 2012). A logistic model was fitted to the data. The choice of the number and shape of trajectories was based not only on the best fit of the model (maximum Bayesian information criteria - BIC) but also on the interpretability of the trajectories obtained (Nagin, 2005).

Multinomial logit models were estimated, relating maternal group membership to predictor variables (maternal socio-demographic and behavioral variables and child´s characteristics), so that the parameters defining the trajectories and the probabilities of trajectory membership were estimated jointly (Nagin, 2005). Individuals with missing information were not excluded from the model due to the ability of group-based trajectory modelling of handling missing data using maximum likelihood estimation (Nagin, 2005).

At the six-year follow-up children were assessed using the Development and Well-Being Assessment (DAWBA) (Goodman et al., 2000), an instrument designed to generate psychiatric diagnosis according to ICD-10 (World Health Organization, 1993) and DSM-IV (American Psychiatric Association, 1994) criteria for ages 5–17 years. The instrument was validated in the Brazilian population (Fleitlich-Bilyk and Goodman, 2004). Trained psychologists administered the DAWBA to mothers or caregivers. The test applied included 12 full sections: separation anxiety disorder, specific phobia, social phobia, generalized anxiety disorder, posttraumatic stress disorder, panic disorder and agoraphobia, obsessive-compulsive disorder, attention deficit hyperactivity disorder (ADHD), oppositional defiant disorder, conduct disorder, eating disorders, and tic disorders. In addition, five screening questions of a previous version of the DAWBA development section were utilized. If any of those were positive, the open questions about development were also asked. Two experienced child psychiatrists reviewed the questionnaires for each child (including free text comments made by respondents) and decided whether to accept or overturn the computer-generated diagnoses. Externalizing disorders included oppositional defiant disorder, conduct disorder and any ADHD, including hyperactive, inattentive and combined sub-types and ADHD not otherwise specified. Internalizing disorders included diagnoses of anxiety and depression.

Family income in the month prior to delivery was collected as a continuous variable in Brazilian Real (BRL). Maternal schooling at the time of delivery was recorded as the number of completed school years of formal education. Maternal age was recorded in complete years. Women who were single, widowed, divorced, or lived without a partner were classified as single mothers. Mother's skin color was self-reported and categorized as white or black/mixed. Parity was defined as the number of previous viable pregnancies and categorized as <2 and ≥2. Maternal depression during pregnancy was defined as “present” if the mother answered positively to question *“During pregnancy, did you feel depressed or have any nervous condition? ”.* Maternal smoking behavior during pregnancy was assessed retrospectively at birth and was self-reported. Regular smokers were those women who smoked at least one cigarette per day on an everyday basis in any trimester of pregnancy. Type of delivery was classified as vaginal or caesarean section.

Birth weight was measured by hospital staff with 10-g precision pediatric scales that were regularly calibrated by the research team. Births below 2500 g were classified as low birthweight. Estimates of gestational age were based on the last menstrual period (LMP) providing they were consistent with predicted birth weight, length, and head circumference, based on the normal curves for these parameters for each week of gestational age (Fenton, 2003). If LMP-based gestational age was unknown or inconsistent, the clinical maturity estimate based on the Dubowitz method (Dubowitz et al., 1970), which was performed on almost all newborns, was adopted. Births before the 37th week of pregnancy were classified as preterm. The type of hospital admission for the newborn after birth was classified as “together with the mother” and “intermediate or intensive care”. At the 3-month follow-up, information on current pattern of breast feeding was collected: exclusive breast feeding, predominant breast feeding (breast milk and herbal teas or water), partial breast feeding (breast milk and other milks – cow's milk or formula – or liquid, semi-solid, or solid food), and weaning (children who were not receiving breast milk).

The child excessive crying, that has been associated with behavioral problems in childhood and maternal mental symptoms (Hemmi et al., 2011, Staehelina et al., 2013, Santos et al., 2015), was assessed at the 3-month visit by asking the mother whether “compared to babies of the same age, her baby cried more, less or as the same”.

The contribution of predictor variables (maternal and child´s characteristics) on bed-sharing trajectory group was examined by using analysis of variance (continuous variables) and chi-square tests (categorical variables). The association between the outcomes (any psychiatric disorder, externalizing and internalizing problems) at age of 6 years and bed-sharing after adjusting for potential confounding factors were analyzed in separate models. Variables were grouped and included in the adjusted analysis using a backward strategy selection. Three models were included for each outcome: unadjusted results (model 1), results adjusted for maternal characteristics (model 2) and results adjusted for model 2 variables plus child´s characteristics (model 3). If the significance level was below 0.20 (Maldonado and Greenland, 1993), the variable remained in the model as a potential confounder for the next level. Interaction terms between maternal trajectories of bed-sharing and gender were tested but not introduced into the model, because they did not reach statistical significance. All analyses were performed with Stata software version 13.0 (StataCorp LP, College Station, Tex).

To deal with residual cofounding related to the low social conditions at which persistent and early-only bed-sharers were born and possibly not completely controlled by means of the multivariable analyses (Rothman et al., 2008), the sample was stratified in two groups: the poorest children (those from the two lowest socio-economic quintiles) and the richest children (those from the two highest socio-economic quintiles). The effect of bed-sharing trajectories over the mental health was analyzed separately for each group.

The study protocol and all follow-ups of the 2004 Pelotas Birth Cohort Study were approved by the Medical Ethics Committee of the Federal University of Pelotas, that is affiliated to the Brazilian National Commission for Research Ethics (CONEP).

## 3 Results

**Fig. 1:**
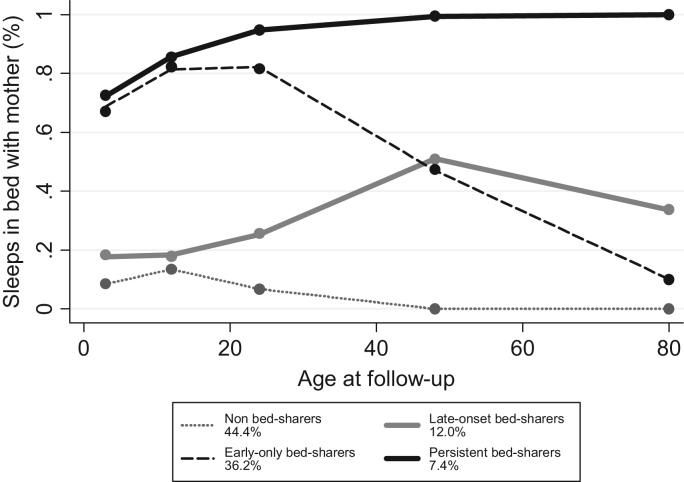
Trajectories of bed-sharing from 3 months to 6 years of age.

The prevalence of bed-sharing at 3, 12, 24, and 48 months of age was 47.8%, 45.9%, 48.3%, and 45.3%, respectively. At 6 years, the prevalence had dropped to 27.4%. Four trajectories of mother-child bed-sharing were identified (Fig. 1): non bed-sharers (44.4%), early-only (36.2%), late-onset (12.0%), and persistent bed-sharers (7.4%).

There were several differences in terms of maternal characteristics between children from different trajectories (Table 1). Non bed-sharers were from wealthier families; their mothers had a higher level of formal education, were generally of white skin color, lived with a partner, and neither smoke nor reported mood depression symptoms during pregnancy.

Early-only bed-sharers (65% of the bed-sharers) bed-shared with their mother until 24 months of age and then steadily reduced that practice until the age of 6 years. Mothers from this group were in average younger and had fewer years of schooling than those from the other three groups.

Late-onset bed-sharers (22% of the bed-sharers) presented a low prevalence of bed-sharing from 3 to 12 months of age and then there was a steadily increase from 12 to 48 months, when about 50% bed-shared with the mother. From 48 months the frequency started to decrease but about 30% of them still bed-shared at the age of 6 years. More than half of the late-onset bed-sharers were born by caesarean section and 30% of the mothers have had birth to two or more children before the index child.

Persistent bed-sharers (13% of the bed-sharers) had a higher prevalence of bed-sharing at the age of 3 months. This prevalence increased until the second anniversary when more than 90% of them bed-shared with the mother. From 24 months to 6 years, there was a plateau in the prevalence. Persistent bed-sharers were in general from poorer families. Their mothers were in average older than mothers from the other three trajectories, 30.5% of them were single, more than one third had non-white skin color, and had a higher parity.

Breastfeeding at three months was more prevalent among early-only (76.7%) and persistent bed-sharers (76.9%) than among non bed-sharers (71.7%).

The prevalence of any psychiatric disorder at the age of 6 years increased from 10.3% among non bed-sharers to 13.2%, 15.8% and 18.8% among late-onset, early-only and persistent bed-sharers, respectively. The prevalence of internalizing problems increased from 7.0–9.3%, 11.5%, and 15.0%, respectively among non bed-sharers, late-onset, early-only and persistent bed-sharers; whereas prevalence of externalizing problems was 3.1%, 3.7%, 5.6%, and 4.5%, respectively.

In the fully adjusted analyses (Table 2), compared to non bed-sharers, persistent bed-sharers presented increased odds of any psychiatric disorder (OR=1.7; 95% CI 1.2–2.5) and of internalizing problems (OR=2.1; 95% CI 1.4–3.1). Among early-only bed-sharers the odds of presenting any psychiatric disorder and internalizing problems were 1.4 (95% CI 1.1–1.8) and 1.6 (95% CI 1.2–2.1), respectively.

Among the poorest children (*n*=1443), prevalence of non bed-sharers, late-onset, early-only and persistent bed-shares was 32.8%, 10.9%, 46.4%, and 10%, respectively (Supplementary Table 1). In the fully adjusted analyses (Table 3), the odds of persistent bed-sharers presenting internalizing problems were three times higher (OR=3.0; 95% CI 1.7–5.5) than that of non bed-sharer children.

Among the richest children (*n*=1429), prevalence of non bed-sharers, late-onset, early-only, and persistent bed-sharers was 56.3%, 13.1%, 25.6%, and 5%, respectively (Supplementary Table 2). In the fully adjusted analyses (Table 4), the odds of bed-sharers present internalizing problems were 70–80% higher than that of non bed-sharers, although not significant.

## 4 Discussion

### 4.1 Trajectories of bed-sharing

This study showed that 56% of the children had the experience of sharing the bed with their mothers regularly at some point of their lives from birth to 6 years of age. Almost two thirds (65%) of those that bed-share were early-only bed-sharers, more than one quarter (22%) were late-onset bed-sharers, and the minority (13%) were persistent bed-sharers).

Bed-sharing practices throughout childhood were described in other studies (Blair et al., 2010, Luijk et al., 2013, Jenni et al., 2005). Similar groups of bed-sharers were found among children from The Avon Longitudinal Study of Parents and Children (ALSPAC), in the United Kingdom (Blair et al., 2010). However, the probability of an infant to share the bed was 3 times higher in Pelotas than in the UK (36.2% versus 13.2% respectively) despite the fact that at the ALSPAC study bed-sharing was defined as an infant or child usually spending some of the nocturnal sleep in the same bed as an adult, not necessarily the mother. There was no association between bed sharing and maternal education or social class among those who constantly shared beds in ALSPAC; whereas in Pelotas children of this group belonged to poorer families and their mothers were more frequently single, smokers and reported depressive symptoms during pregnancy.

At the Generation R Study, in Rotterdam, the Netherlands, sleeping practices were assessed at 2 and 24 months (Luijk et al., 2013). Bed-sharing was defined as the child sharing a bed with the mother or both parents more than three times a week at 2 months and for the majority of the night at 24 months of age: 74% were non bed-sharers, 16% bed-shared at 2 months but not at 24 months, 5% bed-shared only at 24 months, and 5% bed-shared at 2 and 24 months. Single parenthood was associated with all patterns of bed-sharing. Lower education was associated with great odds of persistent and late-onset bed-sharing but not with early-only bed-sharing.

In a study to investigate age trends, long-term course and secular changes of bed-sharing practices and sleep problems among Swiss families, 493 children were followed at 1, 3, 6, 9, 12, 18, and 24 months after birth and at annual intervals thereafter until 10 years of age (Jenni et al., 2005). Parents were queried about bed-sharing during the 3 months before each follow-up interview. Differently from Pelotas, in the first year of life relatively few children slept with their parents (<10%), bed-sharing increased with age and reached a maximum at 4 years, when 38% of the children bed-shared ≥1 times per week. Bed-sharing of at least once per week was noted in 44% of the children between 2 and 7 years old.

### 4.2 Trajectories of bed-sharing and child mental health

In the current study, persistent and early-only bed-sharers presented increased odds of internalizing problems when compared to non bed-sharers. Other studies exploring the effect of bed-sharing on the child behavior and mental health were found at the literature (Madansky and Edelbrock, 1990, Okami et al., 2002, Barajas et al., 2011, Kaymaz et al., 2014). In a randomly selected community sample of 2 year-old children (n =199) and followed-up within 2 weeks of the 3rd anniversary, the majority of parents reported that their child had slept in their bed with them at least once during the previous two months (11% reported that the child always co-slept) (Madansky and Edelbrock, 1990). Bed-sharing was moderately stable over a year with 65% of the initial co-sleepers still co-sleeping one year later. The authors reported only the results of unadjusted analyses. No association between frequent bed-sharing with parents and behavioral problems measured by the Child Behavior Checklist for Ages 2–3 (Achenbach et al., 1987) was found, except for sleep problems that were more common within the frequent bed-sharer group than among the infrequent bed-sharing group.

A cohort of 205 children was followed at birth in 1975 and at the age of 6 years and again at adolescence in 1994 (Okami et al., 2002). Bed-sharing was measured with mothers at the child age of 5 months, 3, 4 and 6 years old (asking about ages 5 and 6 years). Children were then classified in a 5-point continuum from zero (no exposure to bed-sharing) to 4 (frequent exposure): 9% of the parents reported regularly sharing their beds with their 5-month-old infants, 6% at 3, 4 and 5 years, and 3% at age 6 years. There was no indication of any positive or negative effect of bed-sharing in childhood over adolescent outcomes.

To examine the predictors and consequences of mother-child bed-sharing at 1, 2, and 3 years of age, Barajas et al (Barajas et al., 2011), analyzed 944 families with incomes at or below the poverty level, with at least one child younger than 12 months, across the United States. The families were categorized according to how often they reported bed-sharing at these 3 time points: never (52%), 1 time point only (22%), and 2–3 time points (26%). Cognitive (children's math achievement and early literacy skills) and behavioral outcomes (hyperactivity and social skills) were measured at the age of 5 years. Children who bed-shared at more than 1 time point had significantly lower mean scores on social skills than children who never bed-shared. However, the association lost significance once controlled for confounders.

In a small case-control study planned to investigate whether anxiety disorders in adolescents have a link with the separation time of bed/bedroom sharing with parents, Kaimaz et al. (Kaymaz et al., 2014) recruited 51 adolescents who were diagnosed as generalized anxiety disorder with no-comorbidity and 71 healthy adolescents as the control group, who were chosen randomly. Mean duration of bed-sharing in the case group was longer than that of the control group. However, this difference was not statistically significant.

Our results conflict with findings from the other studies. Sample size(Madansky and Edelbrock, 1990, Okami et al., 2002, Kaymaz et al., 2014), lack of adjustment for confounders (Madansky and Edelbrock, 1990). and distinct operational definition of bed-sharing (Madansky and Edelbrock, 1990, Okami et al., 2002, Kaymaz et al., 2014) may have accounted at least in part for the difference between our results and the findings from the other studies.

There is great variability in the prevalence of bed-sharing between and within countries around the world reflecting both methodological issues (divergent methods used in collecting data, different questionnaires and ages of infants) and true intra-population differences in bed-sharing prevalence (Mileva-Seitz et al., 2016). Bed-sharing is said to be one of the parental practices most influenced by cultural practice and beliefs and the effects of bed-sharing over child behavior may depend upon culture. In the United States, for instance, parents of bed-sharing children report greater temperamental problems whereas in other cultures like in Sweden and Japan, with high bed-sharing rates in both infancy and childhood, the rates of emotional and behavioral problems are low (Cassels, 2013).

In Brazil formal recommendations from the part of the Ministry of Health and the Brazilian Pediatric Association in regard to sleep arrangements, are relatively recent and had followed a national educational campaign launched in 2009 by the Children Pastorate advising parents to put infants to sleep on the supine position, as a strategy to prevent the sudden infant death syndrome (Da Ministério Saúde. De Secretaria À Atenção Saúde, 2012, Sociedade Brasileira De Pediatria, 2009). An on-line survey enrolling a national representative sample of Brazilian pediatricians showed that 84.7% of them were aware of the current recommendation of supine sleeping position to prevent the sudden infant death syndrome and that they changed their advice to parents after the educational campaign (Maestri and Nunes, 2016). Before the campaign child sleep practices were seldom discussed even during well-baby clinic consultations and when discussed, mothers were more frequently recommended the lateral and the supine position for newborns (Geib and Nunes, 2006). As a result, in the first years of life of the children from the Pelotas 2004 Birth Cohort, bed-sharing or not bed-sharing was mainly a family issue in Brazil, with bed-sharing being the norm for almost half of the infants (Geib and Nunes, 2006, Santos et al., 2008).

### 4.3 Strengths and limitations

This study is based on data of a cohort that followed children from birth until the age of 6 years with minimal rates of losses. The exposure was assessed at every follow-up so preventing recall bias. The instrument employed for outcome evaluation allows for detection of mental disorders in childhood and showed suitable validity properties when tested in a sample of Brazilian children (Fleitlich-Bilyk and Goodman, 2004).

However, this study did not ask mothers about their reasons for bed-sharing with their children. Some mothers may freely choose to bed-share, while others may do so out of necessity because of household crowding. Some researchers suggest that the outcomes of bed-sharing may depend on whether mothers choose to bed-share (Countermine and Teti, 2010) or whether bed-sharing occurs in reaction to child's sleep problems (Ramos et al., 2007, Sadeh et al., 2010). Mothers that bed-share intentionally and those that bed-share in reaction to a child sleep problem may have a different interpretation of their children behavior (Mileva-Seitz et al., 2016). Although the effect of bed-sharing over the child behavior was adjusted for several covariates including the family socio-economic conditions, maternal mental health and excessive crying of the child, the lack of information on maternal personal reasons for bed-sharing gives place for reverse causality bias and may be considered a limitation of this study.

## 5 Conclusion

This study showed that regular bed-sharing is a common sleeping practice in infancy and childhood in a middle-income setting and that early and persistent bed-sharing is associated with an increased occurrence of internalizing problems at the age of 6 years.

## Contributors

ISS and AM conceived the work, conducted the analyses and draft the first version of the manuscript. TNM and BDPS contributed with the acquisition of data. AJDB and FCB revised the manuscript critically and contributed with interpretation of the findings. All authors approved the submitted version of the manuscript.

## Role of the funding source

The funding sources had no involvement in study design; in the collection, analysis and interpretation of data; in the writing of the report; in preparation of the article; nor in the decision to submit the article for publication.

## Conflict of interest

The authors declare no conflicts of interest.

## Figures and Tables

**Table 1:** Maternal and child characteristics according to bed-sharing trajectories..

Variables	Bed-sharing trajectories				p-value**
	Non bed-sharers *n*=1590	Late-onset bed-sharers *n*=431	Early-only bed-sharers *n*=1296	Persistent bed-sharers *n*=266	
***Maternal characteristics when child was born***					
Family income (Real)*, mean (SD)	1005.9 (1315.7)	866.1 (1180.7)	577.2 (743.7)	503.3 (496.5)	<0.001***
Schooling (years), mean (SD)	9.0 (3.5)	8.7 (3.3)	7.0 (3.1)	7.3 (3.3)	<0.001***
Maternal age (years), mean (SD)	26.8 (6.6)	26.9 (6.9)	24.9 (6.8)	27.6 (7.3)	<0.001***
Single mother, (%)	9.1	15.6	21.5	30.5	<0.001
Skin color, White, (%)	80.9	76.3	64.8	64.7	<0.001
Parity >=2, (%)	30.5	30.4	37.0	44.7	<0.001
Depression symptoms during pregnancy, (%)	20.6	24.0	28.6	29.4	<0.001
Smoking during pregnancy, (%)	20.4	26.7	33.5	36.1	<0.001
C-section, (%)	50.4	52.2	38.8	38.4	<0.001

***Child characteristics***					
Sex, male, (%)	49.8	49.2	54.9	55.3	0.020
Low birthweight, (%)	8.1	9.5	9.5	9.8	0.492
Preterm birth, (%)	12.9	13.7	14.1	16.2	0.479
Intermediate or intensive care hospitalization after birth, (%)	8.9	10.7	8.7	6.0	0.214
Excessive crying at 3 months of age	11.7	13.4	12.2	16.8	0.110
Pattern of breastfeeding at 3 months of age					0.002
Weaning	28.3	27.1	23.3	23.1	
Exclusive breastfeeding	32.9	30.8	30.0	29.6	
Predominant breastfeeding	14.9	19.2	19.4	21.5	
Partial breastfeeding	23.9	22.9	27.3	25.8	

* 1 USD=2.94 Brazilian Real at the year of 2004;

** *x*^2^ test;

*** ANOVA test.

**Table 2:** Crude and adjusted effect of bed-sharing trajectories on psychiatric disorders at 6 years..

Psychiatric disorder		Bed-sharing trajectories				p-value*
		Non bed-sharers OR (IC 95%)	Late-onset bed-sharers OR (IC 95%)	Early-only bed-sharers OR (IC 95%)	Persistent bed-sharers OR (IC 95%)	
Any psychiatric disorder	Model 1^a^	1.0	1.3 (1.0; 1.8)	1.6 (1.3; 2.1)	2.0 (1.4; 2.9)	<0.001
	Model 2^b^	1.0	1.3 (0.9; 1.8)	1.4 (1.1; 1.8)	1.7 (1.2; 2.5)	0.006
	Model 3^c^	1.0	1.3 (0.9; 1.8)	1.4 (1.1; 1.8)	1.7 (1.2; 2.5)	0.007

Internalizing problems	Model 1^a^	1.0	1.4 (0.9; 2.0)	1.7 (1.3; 2.2)	2.3 (1.6; 3.4)	<0.001
	Model 2^b^	1.0	1.3 (0.9; 1.9)	1.5 (1.2; 2.0)	2.1 (1.4; 3.1)	0.001
	Model 3^c^	1.0	1.3 (0.9; 1.9)	1.6 (1.2; 2.1)	2.1 (1.4; 3.1)	0.0008

Externalizing problems	Model 1^a^	1.0	1.2 (0.7; 2.1)	1.8 (1.3; 2.6)	1.5 (0.8; 2.8)	0.014
	Model 2^b^	1.0	1.1 (0.6; 2.0)	1.3 (0.9; 2.0)	1.2 (0.6; 2.2)	0.533
	Model 3^c^	1.0	1.1 (0.6; 2.0)	1.2 (0.8; 1.8)	1.1 (0.6; 2.1)	0.807

a Model 1=crude analysis.

b Model 2=Model 1+maternal characteristics (family income, schooling, age, marital status, skin color, parity, mood depression symptoms and smoking during pregnancy, and type of delivery).

c Model 3=Model 2+child´s variables (sex and excessive crying at the age of 3 months).

**Table 3:** Crude and adjusted effect of bed-sharing trajectories on psychiatric disorders at 6 years of age among the poorest. children (1st and 2nd quintile of family income) (*n*=1443).

Psychiatric disorder		Bed-sharing trajectories				p-value*
		Non bed-sharers OR (IC 95%)	Late-onset bed-sharers OR (IC 95%)	Early-only bed-sharers OR (IC 95%)	Persistent bed-sharers OR (IC 95%)	
Any psychiatric disorder	Model 1^a^	1.0	1.1 (0.6; 1.8)	1.5 (1.1; 2.1)	1.8 (1.1; 3.0)	0.035
	Model 2^b^	1.0	1.1 (0.7; 2.0)	1.4 (1.0; 2.0)	1.8 (1.1; 3.1)	0.084
	Model 3^c^	1.0	1.1 (0.7; 2.0)	1.4 (1.0; 2.1)	2.0 (1.2; 3.4)	0.045

Internalizing problems	Model 1^a^	1.0	1.0 (0.5; 2.1)	1.8 (1.2; 2.8)	2.9 (1.6; 5.1)	<0.001
	Model 2^b^	1.0	1.1 (0.5; 2.2)	1.7 (1.1; 2.7)	2.8 (1.6; 5.1)	0.002
	Model 3^c^	1.0	1.1 (0.6; 2.3)	1.8 (1.2; 2.9)	3.0 (1.7; 5.5)	0.001
					
Externalizing	Model 1^a^	1.0	1.0 (0.4; 2.2)	1.3 (0.8; 2.1)	0.6 (0.2; 1.7)	0.438

problems	Model 2^b^	1.0	1.0 (0.4; 2.3)	1.1 (0.7; 1.9)	0.6 (0.2; 1.7)	0.658
	Model 3^c^	1.0	0.9 (0.4; 2.2)	1.1 (0.6; 1.8)	0.7 (0.2; 1.8)	0.782

a Model 1=crude analysis.

b Model 2=Model 1+maternal characteristics (schooling, age, marital status, skin color, parity, mood depression symptoms and smoking during pregnancy, and type of delivery).

c Model 3=Model 2+child´s variables (sex and excessive crying at the age of 3 months).

**Table 4:** Crude and adjusted effect of bed-sharing trajectories on psychiatric disorders at 6 years among the richest children. (4th and 5th quintile of family income) (*n*=1429).

Psychiatric disorder		Bed-sharing trajectories				p-value*
		Non bed-sharers OR (IC 95%)	Late-onset bed-sharers OR (IC 95%)	Early-only bed-sharers OR (IC 95%)	Persistent bed-sharers OR (IC 95%)	
Any psychiatric disorder	Model 1^a^	1.0	2.0 (1.2; 3.3)	1.9 (1.3; 2.9)	1.7 (0.8; 3.7)	0.002
	Model 2^b^	1.0	1.9 (1.2; 3.1)	1.7 (1.1; 2.5)	1.4 (0.7; 3.1)	0.025
	Model 3^c^	1.0	1.8 (1.1; 3.0)	1.6 (1.1; 2.5)	1.3 (0.6; 2.9)	0.054

Internalizing problems	Model 1^a^	1.0	2.0 (1.2; 3.5)	2.0 (1.3; 3.1)	2.3 (1.1; 5.0)	0.004
	Model 2^b^	1.0	1.9 (1.1; 3.3)	1.7 (1.1; 2.7)	1.9 (0.9; 4.2)	0.042
	Model 3^c^	1.0	1.8 (1.0; 3.1)	1.7 (1.1; 2.7)	1.8 (0.8; 4.0)	0.064

Externalizing problems	Model 1^a^	1.0	1.4 (0.5; 4.5)	3.0 (1.4; 6.5)	0.9 (0.1; 7.4)	0.036
	Model 2^b^	1.0	1.6 (0.5; 5.1)	2.6 (1.2; 5.9)	0.8 (0.1; 6.5)	0.108
	Model 3^c^	1.0	1.6 (0.5; 5.2)	2.2 (1.0; 5.2)	0.7 (0.1; 5.7)	0.227

a Model 1=crude analysis.

b Model 2=Model 1+maternal characteristics (schooling, age, marital status, skin color, parity, mood depression symptoms and smoking during pregnancy, and type of delivery).

c Model 3=Model 2+child´s variables (sex and excessive crying at the age of 3 months).

## References

[bib1] Achenbach T.M., Edelbrock C., Howell C.T. (1987). Empirically based assessment of the behavioral/emotional problems of 2- and 3- year-old children. J. Abnorm. Child Psychol..

[bib2] American Psychiatric Association (1994). Diagnostic and Statistical Manual of Mental Disorder (DSM-IV).

[bib3] Barajas R.G., Martin A., Brooks-Gunn J., Hale L. (2011). Mother-child bed-sharing in toddlerhood and cognitive and behavioral outcomes. Pediatrics.

[bib4] Blair P.S., Heron J., Fleming P.J. (2010). Relationship between bed sharing and breastfeeding: longitudinal, population-based analysis. Pediatrics.

[bib5] Blair P.S., Sidebotham P., Pease A., Fleming P.J. (2014). Bed-sharing in the absence of hazardous circumstances: is there a risk of sudden infant death syndrome? An analysis from two case-control studies conducted in the UK. PLoS One.

[bib6] Cassels T. (2013). ADHD, sleep problems, and bed sharing: future considerations. Am. J. Fam. Ther..

[bib7] Colson E.R., Willinger M., Rybin D., Heeren T., Smith L.A., Lister G., Corwin M.J. (2013). Trends and factors associated with infant bed sharing, 1993–2010: the National Infant Sleep Position Study. JAMA Pedia..

[bib8] Countermine M., Teti D. (2010). Sleep arrangements and maternal adaptation in infancy. Infant Ment. Health J..

[bib9] Da Ministério Saúde. De Secretaria À Atenção Saúde, 2012. Saúde da Criança: Crescimento e Desenvolvimento. Brasília.

[bib10] Das R.R., Sankar M.J., Agarwal R., Paul V.K. (2014). Is “bed sharing” beneficial and safe during infancy? a systematic review. Int J. Pedia..

[bib11] Dubowitz L.M., Dubowitz V., Goldberg C. (1970). Clinical assessment of gestational age in the newborn infant. J. Pedia..

[bib12] Fenton T.R. (2003). A new growth chart for preterm babies: babson and Benda’s chart updated with recent data and a new format. BMC Pedia..

[bib13] Fleitlich-Bilyk B., Goodman R. (2004). Prevalence of child and adolescent psychiatric disorders in southeast Brazil. J. Am. Acad. Child Adolesc. Psychiatry.

[bib14] Geib L.T., Nunes M.L. (2006). Sleeping habits related to sudden infant death syndrome: a population-based study. Cad. Saude Publica.

[bib15] Goodman R., Ford T., Richards H., Gatward R., Meltzer H. (2000). The development and well-being assessment: description and initial validation of an integrated assessment of child and adolescent psychopathology. J. Child Psychol. Psychiatry.

[bib16] Hemmi M., Wolke D., Schneider S. (2011). Associations between problems with crying, sleeping and/or feeding in infancy and long-term behavioural outcomes in childhood: a meta-analysis. Arch. Dis. Child..

[bib17] Horsley T., Clifford T., Barrowman N., Bennett S., Yazdi F., Sampson M., Moher D., Dingwall O., Schachter H., Cote A. (2007). Benefits and harms associated with the practice of bed sharing: a systematic review. Arch. Pedia. Adolesc. Med.

[bib18] Ibge - Instituto Brasileiro De Geografia E Estatística, 2016a. Brasil em sintese [Online]. Available: 〈http://brasilemsintese.ibge.gov.br/populacao/taxas-de-mortalidade-infantil〉 (accessed 25.07.16).

[bib19] Ibge - Instituto Brasileiro De Geografia E Estatística, 2016b. Informações sobre os municípios brasileiros [Online]. Available: 〈http://www.cidades.ibge.gov.br〉 (accessed 25.07.16).

[bib20] Jain S., Romack R., Jain R. (2011). Bed sharing in school-age children--clinical and social implications. J. Child Adolesc. Psychiatr. Nurs..

[bib21] Jenni O.G., Fuhrer H.Z., Iglowstein I., Molinari L., Largo R.H. (2005). A longitudinal study of bed sharing and sleep problems among Swiss children in the first 10 years of life. Pediatrics.

[bib22] Jones, B.L., Naginm, D.S., 2012. A Stata Plugin for Estimating Group-Based Trajectory Models.

[bib23] Kaymaz N., Gokten E.S., Uzun M.E., Yildirim S., Tekin M., Topaloglu N., Binnetoglu F.K. (2014). Prolonged rooming-in in infancy is associated with generalized anxiety disorder in the adolescent period. Int J. Adolesc. Med Health.

[bib24] Luijk M.P., Mileva-Seitz V.R., Jansen P.W., Van I.M.H., Jaddoe V.W., Raat H., Hofman A., Verhulst F.C., Tiemeier H. (2013). Ethnic differences in prevalence and determinants of mother-child bed-sharing in early childhood. Sleep. Med..

[bib25] Madansky D., Edelbrock C. (1990). Cosleeping in a community sample of 2- and 3-year-old children. Pediatrics.

[bib26] Maestri R.N., Nunes M.L. (2016). The uptake of safe infant sleep practices by Brazilian pediatricians: a nationwide cross-sectional survey. Sleep. Med..

[bib27] Maldonado G., Greenland S. (1993). Simulation study of confounder-selection strategies. Am. J. Epidemiol..

[bib28] Mileva-Seitz V.R., Bakermans-Kranenburg M.J., Battaini C., Luijk M.P. (2016). Parent-child bed-sharing: the good, the bad, and the burden of evidence. Sleep. Med. Rev..

[bib29] Mindell J.A., Sadeh A., Wiegand B., How T.H., Goh D.Y. (2010). Cross-cultural differences in infant and toddler sleep. Sleep. Med..

[bib30] Nagin D., Tremblay R.E. (1999). Trajectories of boys' physical aggression, opposition, and hyperactivity on the path to physically violent and nonviolent juvenile delinquency. Child Dev..

[bib31] Nagin D.S. (2005). Group-Based Modeling of Development.

[bib32] Nagin D.S., Odgers C.L. (2010). Group-based trajectory modeling in clinical research. Annu Rev. Clin. Psychol..

[bib33] Ngale K.M., Santos I.S., Gonzalez-Chica D.A., De Barros A.J., Matijasevich A. (2013). Bed-sharing and risk of hospitalisation due to pneumonia and diarrhoea in infancy: the 2004 Pelotas Birth Cohort. J. Epidemiol. Community Health.

[bib34] Okami P., Weisner T., Olmstead R. (2002). Outcome correlates of parent-child bedsharing: an eighteen-year longitudinal study. J. Dev. Behav. Pedia..

[bib35] Prefeitura Municipal De Pelotas, 2016. Available: 〈http://www.pelotas.rs.gov.br〉, (accessed 25.07.16).

[bib36] Ramos K., Youngclarke D., Anderson J. (2007). Parental perceptions of sleep problems among co-sleeping and solitary sleeping children. Inf. Child Dev..

[bib37] Rothman K.J., Greenland S., Lash T.L. (2008). Modern Epidemiology.

[bib38] Sadeh A., Tikotzky L., Scher A. (2010). Parenting and infant sleep. Sleep. Med Rev..

[bib39] Santos I.S., Mota D.M., Matijasevich A. (2008). Epidemiology of co-sleeping and nighttime waking at 12 months in a birth cohort. J. Pedia. (Rio J.).

[bib40] Santos I.S., Matijasevich A., Capilheira M.F., Anselmi L., Barros F.C. (2015). Excessive crying at 3 months of age and behavioural problems at 4 years age: a prospective cohort study. J. Epidemiol. Community Health.

[bib41] Santos I.S., Barros A.J., Matijasevich A., Zanini R., Chrestani Cesar M.A., Camargo-Figuera F.A., Oliveira I.O., Barros F.C., Victora C.G. (2014). Cohort profile update: 2004 Pelotas (Brazil) Birth Cohort Study. Body composition, mental health and genetic assessment at the 6 years follow-up. Int J. Epidemiol..

[bib42] Secretaria Estadual Da Saúde, 2016. Available: 〈http://www.saude.rs.gov.br/〉 (accessed 25.07.16).

[bib43] Seplan - Secretaria Do Planejamento, M.E.D.R., 2016. Atlas Socioeconomico do Rio Grande do Sul [Online]. Available: 〈http://www.atlassocioeconomico.rs.gov.br/〉 (accessed 25.07.16).

[bib44] Sociedade Brasileira De Pediatria, 2009. *Bebês devem dormir de barriga para cima* [Online]. Available: 〈http://www.sbp.com.br/arquivo/bebes-devem-dormir-de-barriga-para-cima/〉 (accessed 25.07.16).

[bib45] Staehelina K., Kurtha E., Schindlera C., Schmidd M., Stutza E. (2013). Predictors of early postpartum mental distress in mothers with midwifery home care – results from a nested case-control study. Swiss Med Wkly.

[bib46] World Health Organization (1993). The ICD-10 Classification of Mental and Behavioural Disorders: Diagnostic Criteria for Research.

