# Supplementary material for: Mother-child bed-sharing trajectories and psychiatric disorders at the age of 6 years
Source: J Affect Disord. 2017 Jan 15;208:163–9. doi: 10.1016/j.jad.2016.08.054 (PMC5159994; doi:10.1016/j.jad.2016.08.054)
Supplement: Supplementary file 1 — Supplementary material [file mmc1.docx]

| Supplementary Table 2. Prevalence of psychiatric disorders at 6 years of age according to bed-sharing trajectories among  the richest children (4^th^ and 5^th^ quintile of family income) (*n*= 1429). | | | | | |
| --- | --- | --- | --- | --- | --- |
| Psychiatric disorder | Bed-sharing trajectories | | | | p-value* |
|  | Non bed-sharers  % (IC 95%)  (n=805) | Late-onset  bed-sharers  % (IC 95%)  (n=187) | Early-only  bed-sharers  % (IC 95%)  (n=366) | Persistent  bed-sharers  % (IC 95%)  (n=71) |  |
| Any psychiatric disorder | 7.7 | 14.4 | 13.9 | 12.7 | 0.002 |
| Internalizing problems | 5.8 | 11.2 | 10.9 | 12.7 | 0.003 |
| Externalizing problems | 1.5 | 2.1 | 4.4 | 1.4 | 0.023 |

* *x*^2^ test
