# Supplementary material for: Mother-child bed-sharing trajectories and psychiatric disorders at the age of 6 years
Source: J Affect Disord. 2017 Jan 15;208:163–9. doi: 10.1016/j.jad.2016.08.054 (PMC5159994; doi:10.1016/j.jad.2016.08.054)
Supplement: Supplementary file 2 — Supplementary material [file mmc2.docx]

| Supplementary Table 1. Prevalence of psychiatric disorders at 6 years of age according to bed-sharing trajectories among  the poorest children (1^st^ and 2^nd^ quintile of family income) (n=1443). | | | | | |
| --- | --- | --- | --- | --- | --- |
| Psychiatric disorder | Bed-sharing trajectories | | | | p-value* |
|  | Non bed-sharers  % (IC 95%)  (n=473) | Late-onset  bed-sharers  % (IC 95%)  (n=157) | Early-only  bed-sharers  % (IC 95%)  (n=669) | Persistent  bed-sharers  % (IC 95%)  (n=144) |  |
| Any psychiatric disorder | 12.1 | 12.7 | 16.9 | 20.1 | 0.035 |
| Internalizing problems | 6.8 | 7.0 | 11.5 | 17.4 | 0.001 |
| Externalizing problems | 5.3 | 5.1 | 6.6 | 3.5 | 0.466 |

* *x*^2^ test
